# Supplementary material for: Circular RNAs in the human brain are tailored to neuron identity and neuropsychiatric disease
Source: Nat Commun. 2023 Sep 18;14:5327. doi: 10.1038/s41467-023-40348-0 (PMC10507039; doi:10.1038/s41467-023-40348-0)
Supplement: Supplementary file 3 — Description of Additional Supplementary Files [file 41467_2023_40348_MOESM3_ESM.pdf]

Description of Additional Supplementary Files

File Name: Supplementary Data 1
Description: Summary for samples and RNAseq experiment.

File Name: Supplementary Data 2
Description: Summary for RNase R treated RNAseq experiments.

File Name: Supplementary Data 3
Description: Cell-specific circRNAs and their host genes.

File Name: Supplementary Data 4
Description: qPCR validation of select circRNAs.

File Name: Supplementary Data 5
Description: Functional enrichment of host genes of all circRNAs and cell-specific circRNAs (BH-adjusted $P < 0.05$, two-sided Fisher's exact test).

File Name: Supplementary Data 6
Description: Diseases enriched for host genes of all circRNAs and cell-specific circRNAs (BH-adjusted $P < 0.05$, two-sided Fisher's exact test).

File Name: Supplementary Data 7
Description: circRNAs differentially expressed in dopamine neurons between ILB and control ($P < 0.05$, two-sided Wald test)
