# Supplementary material for: Circular RNAs in the human brain are tailored to neuron identity and neuropsychiatric disease
Source: Nat Commun. 2023 Sep 18;14:5327. doi: 10.1038/s41467-023-40348-0 (PMC10507039; doi:10.1038/s41467-023-40348-0)
Supplement: Supplementary file 5 — Reporting Summary [file 41467_2023_40348_MOESM5_ESM.pdf]

nature portfolio

Corresponding author(s): Clemens R. Scherzer, MD

Last updated by author(s): Jun 6, 2023

Reporting Summary

Nature Portfolio wishes to improve the reproducibility of the work that we publish. This form provides structure for consistency and transparency in reporting. For further information on Nature Portfolio policies, see our Editorial Policies and the Editorial Policy Checklist.

Statistics

For all statistical analyses, confirm that the following items are present in the figure legend, table legend, main text, or Methods section.

n/a	Confirmed	
☐	☒	The exact sample size (*n*) for each experimental group/condition, given as a discrete number and unit of measurement
☐	☒	A statement on whether measurements were taken from distinct samples or whether the same sample was measured repeatedly
☐	☒	The statistical test(s) used AND whether they are one- or two-sided<br>*Only common tests should be described solely by name; describe more complex techniques in the Methods section.*
☒	☐	A description of all covariates tested
☐	☒	A description of any assumptions or corrections, such as tests of normality and adjustment for multiple comparisons
☐	☒	A full description of the statistical parameters including central tendency (e.g. means) or other basic estimates (e.g. regression coefficient) AND variation (e.g. standard deviation) or associated estimates of uncertainty (e.g. confidence intervals)
☐	☒	For null hypothesis testing, the test statistic (e.g. *F*, *t*, *r*) with confidence intervals, effect sizes, degrees of freedom and *P* value noted<br>*Give P values as exact values whenever suitable.*
☒	☐	For Bayesian analysis, information on the choice of priors and Markov chain Monte Carlo settings
☒	☐	For hierarchical and complex designs, identification of the appropriate level for tests and full reporting of outcomes
☐	☒	Estimates of effect sizes (e.g. Cohen's *d*, Pearson's *r*), indicating how they were calculated

Our web collection on statistics for biologists contains articles on many of the points above.

Software and code

Policy information about availability of computer code

Data collection	All code for the analytical pipeline, including data collection, is available at https://github.com/sterding/circRNA.
Data analysis	All code for the analytical pipeline, including data analysis and software version, is available at https://github.com/sterding/circRNA.

For manuscripts utilizing custom algorithms or software that are central to the research but not yet described in published literature, software must be made available to editors and reviewers. We strongly encourage code deposition in a community repository (e.g. GitHub). See the Nature Portfolio guidelines for submitting code & software for further information.

Data

Policy information about availability of data

All manuscripts must include a data availability statement. This statement should provide the following information, where applicable:

- Accession codes, unique identifiers, or web links for publicly available datasets
- A description of any restrictions on data availability
- For clinical datasets or third party data, please ensure that the statement adheres to our policy

The RNA-seq raw data for the 197 samples (190 brain samples and 7 non-brain samples) is hosted in NIH GEO repository (accession number: GSE218203) and will be publicly available to download. The proceeded data for this study, including a browser track hub for all circRNAs from this study, can be queried at the BRAINcode project website at http://www.humanbraincode.org through a user-friendly interface. Other data supporting the findings of this study are provided in the Supplementary Information/Source Data file

Human research participants

Policy information about studies involving human research participants and Sex and Gender in Research.

Reporting on sex and gender	Findings apply to brains of male and female gender/sex. We included gender as covariate in our analysis of PD-associated circRNA expression. Clinical information on gender was extracted from autopsy reports. For Q/C purposes (sample mishandling), marker expression of the female-specific XIST gene and male-specific Y-chromosome gene RPS4Y1 was evaluated. Source, demographic characteristics, and IRB approval concerning the extant human postmortem brain samples and other biosamples used in this research are reported in detail in the Supplement in Table S1 and in Section S1 Sample Collection and Processing.
Population characteristics	All human samples have information on age of death and clinical diagnosis (see Supplementary Table S1). The 190 brain samples are composed of 102 samples from healthy controls ("HC") subjects, 27 from incidental Lewy body cases ("ILB"; e.g. clinically healthy individuals, found to have PD-associated alpha-synuclein-positive Lewy bodies on autopsy), 18 from Parkinson's disease cases ("PD"), and 43 from Alzheimer's disease cases ("AD"). Details of inclusion and exclusion criteria are described in the Method section.
Recruitment	In our BRAINcode project, we collected 197 high-quality human samples (incl. 190 frozen postmortem human brain samples and 7 non-brain samples) identified from Banner Sun Health Institute, Brain Tissue Center at Massachusetts General Hospital, Harvard Brain Tissue Resource Center at McLean Hospital, University of Kentucky ADC Tissue Bank, the University of Maryland Brain and Tissue Bank, Pacific Northwest Dementia and Aging Neuropathology Group (PANDA) at University of Washington Medicine Center, and Neurological Foundation of New Zealand Human Brain Bank.
Ethics oversight	Institutional Review Board of Brigham and Women's Hospital, Inc.

Note that full information on the approval of the study protocol must also be provided in the manuscript.

Field-specific reporting

Please select the one below that is the best fit for your research. If you are not sure, read the appropriate sections before making your selection.

☒ Life sciences ☐ Behavioural & social sciences ☐ Ecological, evolutionary & environmental sciences

For a reference copy of the document with all sections, see nature.com/documents/nr-reporting-summary-flat.pdf

Life sciences study design

All studies must disclose on these points even when the disclosure is negative.

Sample size	Our study used a large samples size of 197 distinct, high-quality human samples (190 brain and 7 non-brain samples) for total RNAseq analyses. This is orders of magnitude larger than other cell type-specific RNAseq studies. For DE analysis, power analysis was used to estimate sample size requirements.
Data exclusions	Participant inclusion/exclusion criteria are delineated in detail in the Methods section of "Sample Collection and Processing". In details, Inclusion criteria: (1) absence of clinical or neuropathological diagnosis of a neurodegenerative disease e.g., Parkinson's disease according to the UKPDBB criteria, Alzheimer's disease according to NIA-Reagan criteria, dementia with Lewy bodies by revised consensus criteria. (2) PMI ≤ 48 hours; (3) RIN ≥ 6.0 by Agilent Bioanalyzer (good RNA integrity); (4) visible ribosomal peaks on the electropherogram. Exclusion criteria were: (1) a primary intracerebral event as the cause of death; (2) brain tumor (except incidental meningiomas); (3) systemic disorders likely to cause chronic brain damage. Sample exclusion criteria are described in the Supplementary Fig 1 and detailed in the "Sample QC based on RNA-seq data" section of Method.
Replication	RNAseq was replicated using technical and biological replicates (see Rep column in the Supplementary Table 1). circRNAs identified by lcRNAseq were confirmed by a second method, bulk RNAseq on RNase R treated independent brain samples as described in Figure S2. Selected circRNAs were confirmed by qPCR as described in Figure S3.
Randomization	The samples in our study were allocated into clinical groups based on their neurological or neuropathological diagnosis: healthy controls ("HC"), incidental Lewy body cases ("ILB"), Parkinson's disease cases ("PD"), and Alzheimer's disease cases ("AD"). Details of inclusion and exclusion criteria are described in the Method section. In the differential expression analysis between groups, covariates (such as age, sex, PMI, batch) were evaluated and adjusted for in the analysis.
Blinding	When performing laser-capture microdissection RNAseq on brain tissue sections, operators are blinded to diagnosis. When identifying circRNAs on individual RNA-seq sample the analysis is also blinded to the diagnosis. When calling cell-specific circRNAs and diagnosis-associated circRNAs, we are not blind to the diagnosis and cell types, because the group info is the variable of interest in that case.

Reporting for specific materials, systems and methods

We require information from authors about some types of materials, experimental systems and methods used in many studies. Here, indicate whether each material, system or method listed is relevant to your study. If you are not sure if a list item applies to your research, read the appropriate section before selecting a response.

Materials & experimental systems

n/a	Involved in the study	
☒	☐	Antibodies
☒	☐	Eukaryotic cell lines
☒	☐	Palaeontology and archaeology
☒	☐	Animals and other organisms
☒	☐	Clinical data
☒	☐	Dual use research of concern

Methods

n/a	Involved in the study	
☒	☐	ChIP-seq
☒	☐	Flow cytometry
☒	☐	MRI-based neuroimaging
